# Reciprocal interactions between prostaglandin E2- and estradiol-dependent signaling pathways in the injured zebra finch brain

**DOI:** 10.1186/s12974-017-1040-1

**Published:** 2017-12-29

**Authors:** Alyssa L. Pedersen, Colin J. Saldanha

**Affiliations:** Department of Biology, Program in Behavior, Cognition and Neuroscience, and the Center for Behavioral Neuroscience, American University, 4400 Massachusetts Avenue NW, Washington, DC 20016 USA

**Keywords:** PGE2, Estradiol, Brain injury, Astrocytic aromatase

## Abstract

**Background:**

Astrocytic aromatization and consequent increases in estradiol are neuroprotective in the injured brain. In zebra finches, cyclooxygenase-activity is necessary for injury-induced aromatase expression, and increased central estradiol lowers neuroinflammation. The mechanisms underlying these influences are unknown. Here, we document injury-induced, cyclooxygenase-dependent increases in glial aromatase expression and replicate previous work in our lab showing increases in central prostaglandin E2 and estradiol following brain damage. Further, we describe injury-dependent changes in E-prostanoid and estrogen receptor expression and reveal the necessity of E-prostanoid and estrogen receptors in the injury-dependent, reciprocal interactions of neuroinflammatory and neurosteroidogenic pathways.

**Methods:**

Adult male and female birds were shams or received bilateral injections of the appropriate drug or vehicle into contralateral telencephalic lobes.

**Results:**

Injuries sustained in the presence of indomethacin (a cyclooxygenase inhibitor) had fewer aromatase-expressing reactive astrocytes relative to injuries injected with vehicle suggesting that cyclooxygenase activity is necessary for the induction of glial aromatase around the site of damage. Injured hemispheres had higher prostaglandin E2 and estradiol content relative to shams. Importantly, injured hemispheres injected with E-prostanoid- or estrogen receptor-antagonists showed elevated prostaglandin E2 and estradiol, respectively, but lower prostaglandin E2 or estradiol-dependent downstream activity (protein kinase A or phosphoinositide-3-kinase mRNA) suggesting that receptor antagonism did not affect injury-induced prostaglandin E2 or estradiol, but inhibited the effects of these ligands. Antagonism of E-prostanoid receptors 3 or 4 prevented injury-induced increases in neural estradiol in males and females, respectively, albeit this apparent sex-difference needs to be tested more stringently. Further, estrogen receptor-α, but not estrogen receptor-β antagonism, exaggerated neural prostaglandin E2 levels relative to the contralateral lobe in both sexes.

**Conclusion:**

These data suggest injury-induced, sex-specific prostaglandin E2-dependent estradiol synthesis, and estrogen receptor-α dependent decreases in neuroinflammation in the vertebrate brain.

## Background

Estradiol (E_2_) modulates a wide set of morphological and physiological endpoints across the lifespan in many vertebrates. While the influence of E_2_ on various indices of neuroplasticity has long been established [1], there is now an emerging role for this steroid in neuroprotection against degeneration and inflammation following insult to the CNS, including traumatic brain injury (TBI; [2, 3]). Indeed, E_2_ is associated with a decreased risk or progression of a variety of clinical diseases, including Alzheimer’s disease, multiple sclerosis, schizophrenia, and some sleep disorders [4–6], and improves outcomes following experimental TBI and stroke [3, 7–9].

In addition to peripheral sources, the brain itself is capable of E_2_ synthesis via the expression of aromatase (*estrogen*-*synthase*) in neurons of the limbic forebrain [10]. In songbirds and mammals, ischemic or excitotoxic brain injury also induces aromatase expression in reactive astrocytes immediately around the site of damage [3, 11–17]. In songbirds, this induction is particularly rapid, dramatic, and sustained [11, 15, 18, 19] and results in a robust increase of local E_2_ content around the site of injury [20]. Locally derived E_2_ is a potent modulator of cell turnover as it decreases apoptosis and increases cyto- and neurogenesis [14, 15, 18, 21–23]. Glial aromatization also decreases microglial activation following experimental stroke [9] and other indices of inflammatory signaling following brain injury [24].

In recent work, we have posited an association between neuroinflammatory signaling pathways and injury-induced increases in neural aromatization in zebra finches (*Taeniopygia guttata*). This association reflects a feedback loop of inflammatory and neurosteroidogenic signaling in the injured brain and includes two stages: the induction of aromatase expression by injury-induced inflammatory signals and the subsequent anti-inflammatory effects of injury-induced increases in central E_2_. Indeed, inhibition of cyclooxygenase (COX) 1/2-activity with indomethacin mitigates the robust induction of aromatase and E_2_ soon after a penetrating brain injury. More specifically, birds who received indomethacin or vehicle delivered via a penetrating needle into contralateral lobes had lower prostaglandin E2 (PGE2), aromatase-expression, and E_2_ content in the hemisphere injected with the COX-inhibitor [25]. These data strongly suggest that aspects of injury-induced inflammatory signaling are, in part, responsible for the induction of aromatase following brain damage. These increases in central E_2_ exert powerful inhibitory influence on inflammatory signaling. Specifically, birds injected with the aromatase inhibitor fadrozole alone or with concomitant E_2_ demonstrated elevated and decreased COX2 expression and PGE2 content relative to vehicle, respectively [24]. These data strongly suggest an anti-inflammatory role for injury-induced aromatization via the synthesis of E_2_. This unique feedback between neuroimmune and neuroendocrine signaling may serve as a powerful model towards understanding the role of inflammation and steroidogenesis in neuroprotection. Despite these recent findings, the cell-specificity (glial or neuronal) of COX-dependent aromatase expression is unclear, and the mechanisms underlying the interactions of inflammatory and neurosteroidogenic signaling pathways during brain injury are completely unknown. We investigate this in experiment 1 and predict that COX activity increases glial aromatase following brain injury.

PGE2 has a high affinity for four known E-prostanoid (EP) receptors: EP 1-4 [26]. Binding of PGE2 to these receptors can regulate aromatase and E_2_ via modulation of downstream signaling pathways in other systems [27–29]. EP-1 and 2 regulate aromatase in adipose stromal cells [28], while EP-2 and 4 are necessary for the modulation of aromatase in breast cancer cells [29]. However, nothing is known about the association of any EP receptors and aromatase during brain trauma. Further, EP-dependent function is surprisingly understudied in songbirds. This impedes progress in our understanding the interactions between neuroimmune and neurosteroidogenic signaling pathways during brain trauma. Correspondingly, there are three known estrogen receptors (ER): ER-α, ER-β, and the g-protein coupled receptor-1 (GPER1). Of these, ER-α is a potent modulator of cell death and infarct size following experimental stroke with correlated effects on NADPH oxidase activation, cytokine release, and microglia activation and following ischemia or lipopolysaccharide administration [30–33]. ER-α along with ER-β may mediate neurogenesis after ischemia [34], suggesting important roles for ERs in neuroinflammation and cell-turnover. However, which ERs regulate PGE2 and other indices of neuroinflammation during brain injury is unknown, particularly in the songbird. We investigate the role of prostanoid and estrogen receptors in experiment 2 and 3 and predict a receptor(s) will be involved in cox-dependent increases in E_2_ and consequent decreases in PGE2.

Here, we document injury-associated, COX-dependent increases in glial aromatase expression and replicate increases in central PGE2 and E_2_ following penetrating brain injury. Further, we describe injury-dependent changes in PGE2- and ER expression and reveal the necessity of specific EP receptors and ER in the injury-dependent, reciprocal interactions of neuroinflammatory and neurosteroidogenic pathways.

## Methods

### Subjects, housing, and general surgical technique

Adult (> 90 days of age) male and female zebra finches were group housed in same-sex, walk-in aviaries (15–30 per 4 × 6 × 7ft cage) in the animal facility at American University in a humidity (75%) and temperature (77 °F) controlled room with food and water provided ad libitum*.* American University Institutional Animal Care and Use Committee approved all procedures. All experiments used the identical surgical technique in that the injection needle served as the penetrating injury, and the injected reagent served as either the independent variable or control. Subjects served as their own controls with treatments or vehicle controls delivered into contralateral hemispheres. Surgeries were performed in an identical manner according to previously published protocols [14, 15, 18, 24, 25]. Subjects were anesthetized with isoflurane and positioned in a stereotaxic apparatus with the head angled at 45°, the cranium was exposed and bilateral (experiments 1 and 3) or unilateral (experiment 2) craniotomies at 2 mm caudal to the pineal gland, and 3 mm lateral to the midline. Then, a 22-g needle was lowered to 3 mm below the brain surface targeting the entopallial nucleus [14, 15] and remained at this location for 60 s. Depending on the experiment, treatment or vehicle solution was injected into each telencephalic hemisphere over a 60-s duration and the needle left in place for an additional 60 s. The needle was then removed, the cranium swabbed, and the scalp was sealed with Collodion Flexible (EM Sciences, Hatfield, PA).

#### Experiment 1. Inhibition of COX1/2 activity and the induction of aromatase expression in glia

Injury-induced and COX-dependent increases in central aromatase expression and E2 content are detectable more rapidly in females compared to males [19, 25]. Although several studies have established the cellular identity of injury-induced aromatase expression as glial [15, 19], the cell-type responsible for COX1/2-dependent increases in aromatase expression remains unknown; we used an antibody specific to songbird aromatase [35] to reveal and quantify the expression of aromatase around the site of damage following injection of indomethacin or vehicle. Adult zebra finches (*n* = 3 per sex) received bilateral injuries according to previously published protocols [14, 15, 18, 24, 25]. Albeit low, this sample size is compatible with previous studies on injury-induced aromatase expression in astrocytes [11, 14, 15]. During surgery, a 10 μl of a 15 μg/ml solution of indomethacin or 10 μl of vehicle (5% ethanol in 0.9% NaCl) was injected into each telencephalic hemisphere as previously described [25]. Since we were interested in examining the effect of indomethacin on the early stages of aromatase induction, females were euthanized at 6 h, whereas males were euthanized at 24 h post-surgery because injury-induced aromatase expression occurs more rapidly in females [19] and the effect of indomethacin on aromatase expression is detectable earlier in females [25].

At either 6 h (females) or 24 h (males), animals were decapitated, and the brain was rapidly extracted, fixed via immersion in 5% acrolein, gel embedded, and sectioned at 50 μm on a vibratome. Immunocytochemistry (ICC) for aromatase was performed according to previously published protocols [14, 15, 35–37]. Briefly, sections were washed in 0.1 M phosphate buffer (PB), rinsed in H_2_0_2_, and washed again before incubation in normal goat serum. Sections were then placed in primary antibody solution (AZAC; 1:1000) for 72 h. Following incubation, sections were washed overnight, placed in secondary (1:200 biotinylated goat-anti-rabbit IgG) and then incubated in avidin-biotin complex (1:200). Aromatase-immunoproduct was visualized with a peroxide/peroxidase reaction (Vector SG, Burlingame, CA). Sections were then mounted on slides and coverslipped following dehydration through graded alcohols [35]. Slides were examined on a Nikon eclipse E100M at ×100 (to locate the area of injury-induced aromatase expression and ×400 (to more stringently verify the cellular identity of injury-induced aromatase expression) magnification. The area around the injury was imaged, and the injury was recognized as a dorsal to ventral tear or a hole in the tissue. In order to sample the density of immunoreactive cells, sections were examined at ×400, the injury tract was moved just off the frame, and images in which the cells were distributed over the entire frame were captured. Two images were collected per hemisphere/treatment for a total of four images per bird (total of 24 images). All images were coded, and an experimenter who was blind to treatment conditions counted the number of cells in each image. The total number of labeled cells was counted, averaged within hemisphere, and compared statistically across treatment condition.

#### Experiment 2. Change in PGE2 and E2 receptors following penetrating brain injury

To examine the changes in E-prostanoid (EP 2-4) and estrogen receptors (ER-α, ER-β, and GPER1) following injury, male and female zebra finches received a unilateral penetrating brain injury according to previously published protocols [14, 24] and were sacrificed either 2-, 6-, or 24-h post-surgery (*n* = 5 of each sex per time point). At each time-point, subjects were decapitated and the telencephalic lobes were rapidly dissected into four quadrants [24]. The posterior quadrants (where the injury was located) were used for experiments. Samples were homogenized in 500 μL of phosphate buffer, and 100 μL of homogenate were used for qPCR analyses to measure prostanoid and estrogen receptors. The remaining homogenate was stored at − 80 °C for future analyses.

### Quantitative polymerase-chain reaction

RNA extraction, cDNA synthesis, and qPCR were performed according to previously published protocols from our lab [24, 25]. Briefly, 100 μL of homogenate was used to isolate RNA using RNeasy Mini Extraction Kit (Qiagen, Germantown, MD) according to manufacturer’s instructions. One microgram of total RNA from each sample was reversed transcribed using the high capacity cDNA Reverse Transcription Kit (Life Technologies, Carlsbald, CA). Five microliters of the resulting cDNA was used to perform RT-qPCR using SYBR Select Master Mix (Life Technologies, Carlsbald, CA). Primers for EP 2-4 were generated against the zebra finch genome and validated in our lab (EP-1 does not appear in the zebra finch genome, see Table 1 for sequences and Accession numbers). Previously validated primers for ER-α, ERβ, and GPER1 were generated against the zebra finch genome and used in this study.

**Table 1:** List of primers used for amplification using qPCR

Gene	Accession number	Forward primer	Reverse primer
EP-1	N/A	N/A	N/A
EP-2	XM012573981	^1^GAGATGGAGGAGGGAGTGCG ^2^GGGGTGGATTCGTCATCCGT	^1^GAAGACCCAGGGATCCACGA ^2^TGGCGTATAGCACGGGGAAG
EP-3	XM002187017.3	CCCGTCGTCATCTCCGTGTA	AGCGTCATGCTGAAGCCGAA
EP-4	XM012577596.1	CGCATTGCCTCAGTGAACC	GCCACCAGAGCTGATTTCGC
ER-α	NM001076701	TCGCCCTTCATCCATCATCACA	TGTGGCGCCTGTTAT CGG AGTT
ER-β	XM002200595	TGGTCCTGTGAAGGCTGCAA GTC	TGCGCCGGTTTTTGTCTA TTGTG
GPER	XM004175666	GGCTVTCGCCATGATTTTTGTTG	CATGCC TGAAGGATG GGCTGTT
PiK3	XM002191515.2	GGAGACAAAGAAGTGACTGGAAGCC	TCCTCTGAGCTCTGCACTTCTTGA
PKA	XM002196441.3	GGCAGGGGGTTGGAAGTTGA	TGCACCTGGCACCATCTCTT

#### Experiment 3. Antagonism of estrogen or prostanoid receptors and downstream interactions with PGE2 and E_2_ synthesis

In previous studies, we have found that COX1/2 activity is necessary for injury-induced increases in aromatase and E_2_ content [25]. These increases in local E_2_ have anti-inflammatory effects as inhibition of injury-induced aromatization, and E_2_ replacement exacerbates and mitigates neural cytokines and PGE2 content [24]. The mechanisms that underlie the effects of PEG2 receptors on aromatase activity and those that underlie the effects of E_2_ on inflammation remain unknown. Based on preliminary data (see below), a total of four receptors were targeted. Two prostanoid receptors (EP-3 and EP-4) were antagonized to investigate the role of these receptors in the upregulation of E_2_. Two estrogen receptors, ER-α and ER-β, were targeted as possible candidates for the anti-inflammatory effects of E_2_ following injury to the brain. In this study, animals were sacrificed at 24 h, based on a previous study in our lab [24].

### Surgery

To investigate the role of prostanoid receptors in the upregulation of E_2_, male and female zebra finches served as anesthetized but otherwise unmanipulated shams (*n* = 5/sex) or received bilateral injuries where contralateral hemispheres received injections of vehicle (5% ethanol in 0.9% NaCl) or specific receptor antagonists (*n* = 10/sex). Ten microliters of a 10 μg/ml of an EP-3 antagonist (L-798, 106; *n* = 10), or an EP-4 antagonist (BCG-20-1531 hydrochloride, *n* = 10), was injected during injury to one hemisphere. Doses were based upon previously published protocols using these antagonists in avian and rodent models [38–42]. Because of the aforementioned sex-difference in injury-induced, COX-dependent increase in aromatase expression, females were sacrificed at 6 h and males at 24 h post-surgery.

To investigate the role of ERs in the anti-inflammatory influence of E_2_, male and female zebra finches served as anesthetized but otherwise unmanipulated shams (*n* = 5/sex) or received bilateral injuries with contralateral hemispheres received injections of vehicle (5% ethanol in 0.9% NaCl) or specific receptor antagonists (*n* = 10/sex). Ten microliter of a 10-μg/ml solution of an ER-α antagonist (MMP) or an ER-β antagonist (PHTPP) was injected during injury into one hemisphere. As mentioned above, doses were chosen based on previously published data in avian and rodent models [38–42]. All subjects were sacrificed 24 h post-surgery, a time point where the anti-inflammatory effects of injury-induced E_2_ are clearly observable [24]. Following euthanasia, telencephalic lobes were rapidly dissected into four quadrants, and the cerebellum and anterior quadrants were discarded, leaving only the area surrounding the injury. Posterior quadrants were weighed and homogenized in 500 μL of phosphate buffer and then divided into two aliquots: 100 μL for qPCR and 300 μL for EIA.

### Quantitative polymerase-chain reaction

To test if receptors were successfully antagonized, we measured a target downstream of the receptor. For prostanoid receptors, we measured protein kinase A (PKA) mRNA, and for estrogen receptors, we measured phosphoinositide-3-kinase (PiK3). Both PKA and PiK3 have been routinely shown to be downstream of prostanoid or estrogen receptors [43–47]. Importantly, previous studies have measured the mRNA for these targets in the periphery and in the brain, suggesting that it is a reliable measurement [48–50]. qPCR was done identically to the procedure above and according to previously published protocols [24, 25]. Primers for PKA and PiK3 were generated against the zebra finch genome (see Table 1) and validated in our lab.

### PGE2 enzyme immunoassay sample preparation and enzyme immunoassay

Three hundred microliters of homogenate was used for a combined solid and liquid phase extraction according to previously published protocols from our lab [20, 25, 51, 52]. On the day of EIA assay, samples were assayed in triplicates using a commercial PGE2 EIA kit (Cayman Chemical, Ann Arbor, MI) that has been previously validated for zebra finch brain tissue [24]. Before ether and solid-phase extraction, an additional sample was spiked with radio-inert PGE2 to the concentration of 125 pg/mL to estimate recovery. The remaining reconstituted sample was placed at − 80 °C until the day of E_2_ assay (see below).

### E_2_ enzyme immunoassay

The remaining reconstituted sample (≈ 200 μL) from the PGE2 assay was used for the E_2_ EIA (Cayman Chemical, Ann Arbor, MI) that has been previously validated with zebra finch brain tissue [20, 51, 53]. The remaining sample was removed on the day of the assay and further diluted with EIA buffer. Similar to the PGE2 assay, two samples were spiked with E_2_ to the concentration of 256 pg/mL to estimate recovery and ran alongside experimental samples. Blank wells were included on each assay plates (PGE2 and E_2_) to assess the possibility of containments. Blank wells gave an average read of 0.002.

### Statistics

#### Experiment 1. Inhibition of COX1/2 activity and the induction of aromatase expression in glia

To assess if indomethacin treatment affected glial aromatase expression following brain injury, we performed two one-way analysis of variance (ANOVAs) with treatment as the main variable, which was coded as “within subject.” Sex was analyzed separately due to males and females being euthanized at different time points.

#### Experiment 2. Change in PGE2 and E2 receptors following penetrating brain injury

In order to assess the changes in prostanoid (EP 2-4) and estrogen receptors (ER-α, ER-β, and GPER1) following unilateral brain injury, and in order to increase the stringency of our analyses, we performed two three-way analysis of variance (ANOVAs) on ΔCT values with gene, time, and treatment as main variables, and treatment coded as “within subject.” Sexes were analyzed separately. The source of significant main effects was queried using Tukey-Kramer posthoc analysis, and significant interactions were assessed with Fisher LSD pairwise comparisons.

#### Experiment 3. Antagonism of estrogen or prostanoid receptors and downstream interactions with PGE2 and E_2_ synthesis

All three measures (qPCR, PGE2 EIA, and E_2_ EIA) were analyzed with a one-way nested model ANOVA. Treatment included sham animals or animals that underwent surgery (antagonist or vehicle in contralateral hemispheres). Antagonist/vehicle was coded as “within subject” and nested within the main variable of “treatment.” The source of significant main effects was queried using Tukey-Kramer posthoc analysis, and significant interactions were assessed with Fisher LSD pairwise comparisons. For this experiment, sex was analyzed separately due to the difference in aromatase induction patterns and therefore time at which animals were euthanized (females at 6 h and males at 24 h). For qPCR data, statistical analyses were conducted on the delta threshold cycle number (ΔCT values) method of quantification, but data are presented in fold change. For both the PGE2 and E_2_ EIA, data was converted to picogram/milligram to account for brain weight and statistical analyses were conducted on these numbers.

#### Estrogen receptors

All three measures were analyzed with a two-way nested model analysis of variance with sex and treatment as main variables. Treatment included sham animals or animals that underwent surgery (antagonist or vehicle). Surgery animals were coded as “within subject” and nested within the main variable of “treatment.” The source of significant main effects was queried using Tukey-Kramer posthoc analysis, and significant interactions were assessed with Fisher LSD pairwise comparisons. Since animals were euthanized at the same time point (24 h), we included sex as a variable in our analysis. qPCR data was analyzed by the ΔCT quantification method, but figures are presented as fold change. PGE2 and E_2_ data was obtained in picogram/milliliter and transformed to picogram/milligram to account for brain weight, and analyses were conducted on these values.

## Results

### Experiment 1. Inhibition of COX1/2 activity and the induction of aromatase expression in glia

**Fig. 1:**
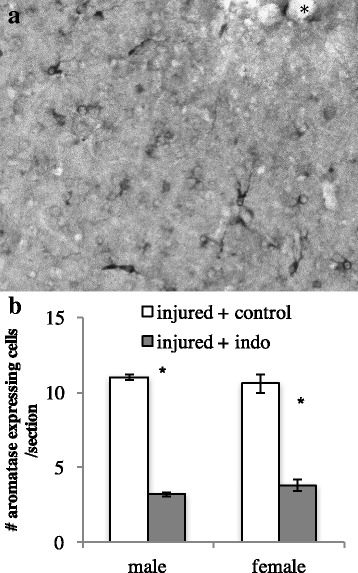
**A** Photomicrograph taken under a ×40 objective lens of aromatase expression in astrocytes around the site of brain injury (asterisk) in the zebra finch. Treatment with indomethacin lowered the number of aromatase-expressing astrocytes relative to injuries treated with vehicle alone (**B**)

Indomethacin decreases glial aromatase following brain injury. Neuronal aromatase was detectable in several telencephalic and diencephalic nuclei across the brain [35], and glial aromatase was detectable around the site of injury, regardless of treatment. In the entopallium, a visual nucleus targeted in the present study, aromatase-expressing cells appear to be astrocytes based upon their large unstained nuclei and numerous short, stellate processes. This is starkly different from the aromatase-expressing neurons in brain areas such as the ventromedial nucleus of the hypothalamus (VMN) where cells are smooth, fusiform, and uni- or bipolar with long processes. We found that treatment with indomethacin decreases glial aromatase as statistical comparisons reveal a main effect of treatment in males (*F* (1, 4) = 37.1, *p* < 0.01) and females (*F* (1, 4) = 136.0, *p* < 0.01) with lower numbers of aromatase-expressing astrocytes around injury sites injected with indomethacin relative to vehicle. See Fig. 1.

### Experiment 2. Change in PGE2 and E2 receptors following penetrating brain injury

#### Prostanoid receptors

We were not able to design primers specific for EP-1 because it is not represented in the zebra finch genome. Using RNA extracted from injured and control brain tissue, we found that EP-2 amplified, but at very low levels (CT value mean = 37.22). Two different sets of primers were designed, and template and primer concentrations were varied and each obtained similar results; as did amplification from liver RNA. Identical experimental conditions readily amplified other gene products. Therefore, further analyses focused on EP-3 and EP-4. ΔCT values are presented in Table 2.

**Table 2:** ΔCT ± SEM of prostanoid and estrogen receptors

	EP-2	EP-3	EP-4	ER-α	ER-β	GPER
Males (uninjured vs. injured)						
2 h	24.0 ± .0.47 vs. 23.59 ± 0.21	10.80 ± 0.70 vs. 11.20 ± 0.78	10.94 ± 0.40 vs. 11.32 ± 0.41	16.27 ± 0.35 vs. 15.83 ± 0.72	13.90 ± 0.41 vs. 14.16 ± 0.35	10.70 ± 0.41 vs. 10.80 ± 0.32
6 h	24.56 ± 0.39 vs. 23.31 ± 0.35	11.50 ± 0.20 vs. 11.85 ± 0.03	*12.32 ± 0.40* vs. *9.84 ± 0.80*	*18.77 ± 0.56* vs. *15.94 ± 0.24*	16.64 ± 0.55 vs. 15.08 ± 0.39	9.98 ± 0.09 vs. 8.72 ± 0.32
24 h	20.97 ± 0.18 vs. 24.25 ± 0.09	11.88 ± 0.20 vs. 12.10 ± 0.29	10.51 ± 0.39 vs.11.33 ± 0.50	*17.42 ± .24* vs. *15.34 ± 0.79*	13.33 ± 0.53 vs. 13.65 ± 0.39	10.96 ± 0.22 vs.10.74 ± 0.29
Females (uninjured vs. injured)						
2 h	21.81 ± 0.36 vs. 23.41 ± 0.97	10.67 ± 0.56 vs. 10.46 ± 0.39	12.30 ± 0.52 vs. 12.74 ± 0.18	15.58 ± 0.79 vs. 15.53 ± 0.34	14.32 ± 0.45 vs. 14.71 ± 0.08	10.80 ± 0.35 vs. 11.65 ± 0.83
6 h	19.3 ± 0.76 vs. 21.66 ± 0.31	*12.73 ± 0.10* vs. *11.86 ± 0.23*	*10.91 ± 0.34* vs. *9.81 ± 0.34*	*18.88 ± 0.74* vs. *15.85 ± 0.78*	*15.54 ± 0.54* vs. *13.04 ± 0.61*	9.51 ± 0.16 vs. 8.94 ± 0.30
24 h	19.82 ± 0.81 vs. 23.85 ± 0.87	12.06 ± 0.14 vs. 12.28 ± 0.17	*10.62 ± 0.15* vs. *8.91 ± 0.07*	16.35 ± 0.86vs. 15.54 ± 0.27	*15.05 ± 0.29* vs. *13.91 ± 0.26*	11.61 ± 0.16 vs. 11.32 ± 0.32

Values in italics indicated significance injured vs. control (*p* < 0.05)

#### Males

Injury to the brain increases EP-4 receptor expression. Analyses revealed a main effect of gene (*F* (1, 24) = 16.92, *p* < 0.01), time (*F* (2, 24) = 3.41, *p* = 0.04), and interactions of gene × time (*F* (2, 24) = 11.0, *p* < 0.01), and gene × time × treatment (*F* (2, 24) = 3.43, *p* = 0.04). No other sources of significance were found (treatment: (*F* (2, 24) = 0.03, *p* = 0.84); gene × treatment: (*F* (1, 24) = 1.72, *p* = 0.20); time × treatment: (*F* (2, 24) = 3.22, *p* = 0.06). Sources of significance are driven by injured hemispheres having higher EP-4 receptor mRNA expression at 6 h, but not at 2 or 24 h. See Table 2 for ΔCT means + SEM from experiment 1.

#### Females

Following injury to the brain, EP-3 and EP-4 mRNA increases in females. Analyses revealed a main effect of gene (*F* (1, 24) = 6.10, *p* = 0.02), treatment (*F* (1, 24) = 7.35, *p* = 0.01), and interactions of variables gene × time (*F* (2, 24) = 18.5, *p* < 0.01) and gene × time × treatment (F (2,24) = 3.71, *p* = 0.03). No other sources of significance were found (time: (*F* (2, 24) = 1.10, *p* = 0.35); gene × treatment (*F* (1, 24) = 1.61, *p* = 0.20); time × treatment (*F* (2, 24) = 2.92, *p* = 0.07)). The sources of significance are due to increased EP-3 receptor mRNA at 6 h post injury, and not at 2 or 24 h. Similarly, brain injury causes EP-4 receptor mRNA to increase at 6 and 24 h, but not at 2 h.

### Estrogen receptors

#### Males

Following brain injury, there was a main effect of gene (*F* (2, 36) = 155.0, *p* < 0.01), treatment (*F* (1, 36) = 8.41, *p* < 0.01), and a significant interaction of gene × time (*F* (4, 36) = 10.60, *p* < 0.01), with no other sources of interaction (time: (*F* (2, 36) = 1.97, *p* = 0.15); gene × treatment: (*F* (2, 36) = 1.81, *p* = 0.17); time × treatment: (*F* (2, 36) = 2.00, *p* = 0.15); gene × time × treatment: (*F* (4, 36) = 0.38, *p* = 0.82)). The main effect of gene and treatment is driven by brain injury increasing ER-α but not ER-β or GPER1 mRNA. The gene × time interaction suggests that there are increases in ER-α mRNA at 6 and 24 h post injury, but not at 2 h. There were no increases in GPER1 mRNA at any time point. See Table 2 for ΔCT means + SEM from experiment 1.

#### Females

Following brain injury, there is a main effect of gene (*F* (2, 36) = 141.0, *p* < 0.01), treatment (*F* (1, 36) = 17.4, *p* < 0.01), and interactions of gene × time (*F* (4, 36) = 4.36, *p* < 0.01), gene × treatment (*F* (2, 36) = 5.38, *p* < 0.01), and a time × treatment (*F* (2, 36) = 4.66, *p* < 0.01). No other sources of significance were found (time: (*F* (2, 36) = 0.90, *p* = 0.41); gene × time × treatment (*F* (4, 36) = 1.30, *p* = 0.28)). The sources of significance are due to increases in ER-α mRNA at 6 h post injury, but not at 2 or 24 h. Brain injury also caused a change in ER-β mRNA at 6 and 24 h, but not at 2 h. There were no changes detected in GPER1 mRNA at any time point.

### Experiment 3. EP-receptors and ER in injury-induced aromatization and inflammation

#### Prostanoid receptors

Given the above data, we chose to target EP-3 and EP-4 to test if antagonism of these receptors would affect injury-induced E_2_. First, we measured central PGE2 levels to ensure there was still a PGE2 induction, regardless of receptor antagonism. Next, we measured PKA mRNA to test if we were inhibiting the receptor resulting in decreased downstream signaling effects. Finally, we measured E_2_ to assess what receptor is necessary for the induction of E_2_ following injury.

### PGE2 EIA

#### EP-3 antagonist

**Fig. 2:**
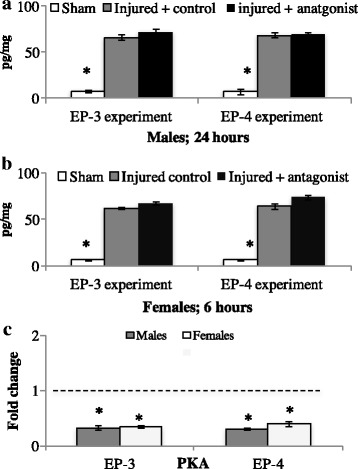
Central levels of prostaglandin E2 (PGE2) in sham animals or following bilateral injury with prostanoid receptor antagonism in adult male and female zebra finches (**A** and **B**). Treatment with EP-3 or EP-4 antagonist during brain injury does not affect the induction of PGE2 (**A** and **B**) compared to sham controls. However, receptor antagonism decreases downstream signaling (PKA) compared to control-injured brains (**C**). Dashed line represents injured controls (controls set to 1 for fold change calculation). *p < 0.05

Following injury to the brain, there was an increase in central PGE2 levels regardless of treatment. As such, there was a main effect of treatment in both males (*F* (2, 8) = 169.0, *p* < 0.01) and females (*F* (2, 8) = 549.0, *p* < 0.01). Overall, shams had the lowest levels of PGE2, and injured hemispheres had elevated PGE2, regardless of treatment or sex. See Fig. 2A.

#### EP-4 antagonist

Similar to EP-3 data, brain injury, regardless of treatment, increased PGE2 levels in both sexes. There was a main effect of treatment in males (*F* (2, 8) = 223, *p* < 0.01) and females (*F* (2, 8) = 313.0, *p* < 0.01). Overall, shams had low levels of PGE2, where injured control and injured EP-4 antagonist hemispheres had higher levels of PGE2.

### qPCR for PKA

#### EP-3 antagonist

Following EP-3 antagonism, there was a main effect of treatment in both males (*F* (1, 4) = 50.3 *p* < 0.01) and females (*F* (1, 4) = 81.8, *p* < 0.01). The main effect is due to both males and females having decreased PKA signaling following EP-3 antagonism compared to controls. See Fig. 2B.

#### EP-4 antagonist

Antagonism of EP-4 also decreased PKA mRNA. As such, there was a main effect of treatment for both males (*F* (1, 4) = 77.8, *p* < 0.01) and females (*F* (1, 4) = 36.9, *p* < 0.01).

### E_2_ EIA

#### EP-3 antagonist

**Fig. 3:**
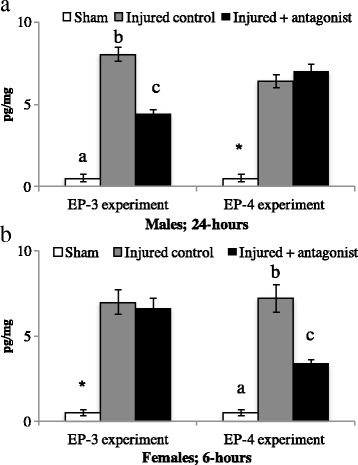
Central levels of estradiol (E_2_) content in sham animals or following bilateral brain injury in adult male and female zebra finches. Males, but not females, fail to induce E_2_ following EP-3 antagonism (**A**). Females, but not males, fail to induce E_2_ following EP-4 antagonism (**B**). Groups that do not share a letter are significantly different (*p* < 0.05)

EP-3 antagonism decreased the induction of E_2_ following injury, but only for males and not females. In males, analyses revealed a main effect of treatment (*F* (2, 8) = 150.0, *p* < 0.01). Overall, shams had the lowest levels of E_2_, and injured control hemispheres had high injury-induced E_2_ content. However, treatment with an EP-3 antagonist decreased E_2_ content following injury. In females, there was also a main effect of treatment (*F* (2, 8) = 47.4, *p* < 0.01). Similar to males, shams had low levels of E_2_. In contrast to males, both injured lobes, regardless of treatment, had injury-induced increases in E_2_. See Fig. 3.

#### EP-4 antagonist

In contrast to EP-3 data, EP-4 antagonism decreases injury-induced E_2_ but only for females. In males, there was a main effect of treatment (*F* (2, 8) = 86.4, *p* < 0.01). The main effect is driven by shams having the lowest levels of E_2_, and both injured hemispheres, regardless of treatment, have increased E_2_ content. For females, there was also a main effect of treatment (*F* (2, 8) = 41.8 *p* < 0.01). Similar to above data, shams have the lowest levels of E_2._ However, only control-injured females show a robust increase in E_2_ content. EP-4 antagonism decreases E_2_ content following brain injury in females.

#### Estrogen receptors

Given the above data (experiment 2), we antagonized ER-α and ER-β to test if they are necessary for the reduction of PGE2 following brain injury. First, we measured central E_2_ levels to ensure there was a robust E_2_ induction following brain injury, regardless of receptor antagonism. Next, we measured PiK3 mRNA to test if antagonism of these receptors was successful and limited downstream signaling. Finally, we measured PGE2 levels to test what receptor is responsible for the anti-inflammatory actions of E_2_ following brain injury.

### E_2_ EIA

#### ER-α antagonist

**Fig. 4:**
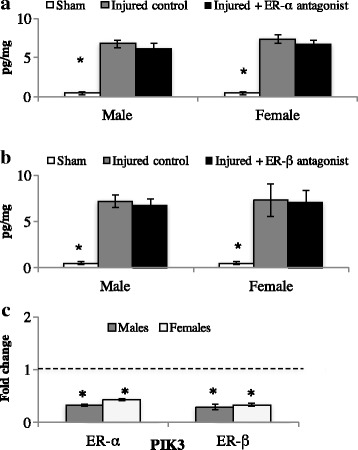
Central levels of estradiol (E_2_) content in sham animals or following bilateral brain injury with estrogen receptor antagonism in adult male and female zebra finches (**A** and **B**). Treatment with an ER-α or ER-β antagonist during brain injury results in robust E_2_ synthesis compared to sham controls (**A** and **B**). However, receptor antagonism decreases downstream signaling (phosphoinositide-3-kinase) compared to control-injured brains. Dashed line represents injured controls (controls set to 1 for fold change calculation). *p < 0.05

Brain injury increases E_2_ content regardless of treatment. There was a main effect of treatment (*F* (1, 24) = 81.0, *p* < 0.01), with no other sources of significance (sex: (*F* (1, 24) = 0.78, *p* = 0.38); sex × treatment: (*F* (2, 24) = 0.19, *p* = 0.82)). Overall, shams had the lowest levels of E_2_. Injured hemispheres have increased E_2_ content regardless of treatment. See Fig. 4A.

#### ER-β antagonist

Brain injury increases central E_2_ content. There was a main effect of treatment (*F* (1, 24) = 20.9, *p* < 0.01), with no other sources of significance (sex: (*F* (1, 24) = 0.02, *p* = 0.87); sex × treatment: (*F* (2, 24) = 0.09, *p* = 0.99). Identical to ER-α antagonist data, shams had the lowest levels of E_2_, and injured hemispheres had increases in E_2_ content, regardless of treatment.

### qPCR for PiK3

#### ER-α antagonist

Antagonism of ER-α decreased PiK3 mRNA. There was a main effect of treatment (*F* (1, 8) = 29.9, *p* < 0.01), with no other sources of significance (sex: (*F* (1, 8) = 2.44, *p* = 0.15); sex × treatment: (*F* (1, 8) = 0.52, *p* = 0.48). See Fig. 4B.

#### ER-β antagonist

Antagonism of ER-β decreased PiK3 mRNA. There was a main effect of treatment (*F* (1, 8) = 18.8, *p* < 0.01), with no other sources of significance (sex: (*F* (1, 8) = 2.82, *p* = 0.13); sex × treatment: (*F* (1, 8) = 0.66, *p* = 0.43).

### PGE2 EIA

#### ER-α antagonist

**Fig. 5:**
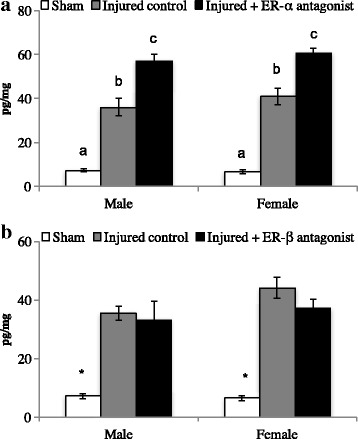
Central levels of PGE2 content in sham animals or following bilateral brain injury with estrogen receptor antagonism in adult male and female zebra finches. ER-α antagonism results in increased PGE2 levels compared to control injured brains (**A**). No such effect was detected following ER-β antagonism (**B**). Groups that do not share a letter are significantly different (*p* < 0.05)

ER-α antagonism increased central PGE2 levels. Results show a main effect of treatment (*F* (1, 24) = 22.6, *p* < 0.01), with no other sources of significance (sex: (*F* (1, 24) = 1.68, *p* = 0.20); sex × treatment: (*F* (2, 24) = 0.78, *p* = 0.48). Post-hoc analyses revealed that shams had low levels of PGE2, and there was an induction of PGE2 following brain injury. However, hemispheres treated with an ER-α antagonist had the highest levels of PGE2. See Fig. 5.

#### ER-β antagonist

Injury to the brain increases PGE2 levels, with no treatment differences. There was a main effect of treatment (*F* (1, 24) = 51.0, *p* < 0.01), with no other sources of significance (sex: (*F* (1, 24) = 1.93, *p* = 0.17); sex × treatment: (*F* (2, 24) = 0.89, *p* = 0.42). Similar to previous data, shams had the lowest levels of PGE2. Brain injury increased central PGE2, with no effect of treatment. Thus, ER-β antagonism does not result in increased PGE2 signaling.

## Discussion

Earlier reports suggest that indices of inflammation including prostaglandin signaling may be both necessary and sufficient for the increases in neural aromatase expression and E_2_-content following penetrating brain damage in the songbird [25, 37]. While other reports strongly support an inductive role for brain damage on astrocytic aromatase expression in songbirds [14, 15, 18, 19, 37, 54] and mammals [6, 55–57], the specific role of COX-activity on aromatase expression in this cell type was unclear. Moreover, the mechanism(s) that supported the inductive role of COX1/2-dependent signaling on aromatization were completely unknown. The present data suggest that glial aromatase is potently affected by local COX1/2 activity following brain damage as evidenced by lower numbers of aromatase-expressing cells of astrocytic morphology around damage associated with indomethacin administration relative to controls. As previous work using double-label immunocytochemistry has established the astrocytic nature of aromatase-expressing cells following mechanical damage [15] and neuroinflammation [37], we are confident that aromatization affected by indomethacin is likely expressed in reactive astrocytes around the site of damage.

The majority of the current set of studies focused on the mechanisms responsible for the reciprocal interactions of injury-associated inflammatory signaling and neurosteroidogenesis. Specifically, given the inductive role of COX-activity on neural aromatization [25] and the anti-inflammatory influence of injury-induced E_2_-synthesis [24], we were interested in how PGE2 may influence neural aromatization following brain trauma and how locally generated E_2_ may regulate neuroinflammation.

### Role of prostanoid receptors on injury-induced aromatization

In a preliminary study, we first sought to describe injury-induced changes in the four known prostanoid receptors (EP 1-4) in the songbird brain using qPCR for these target gene-products at various times following injury. Surprisingly, EP-1 and EP-2 receptors were not represented in the zebra finch genome [58] or could not be amplified with two different sets of primers specific to EP-2 under conditions that revealed abundant and specific amplification of other products from the identical cDNA template. The expression of both EP-3 and EP-4 changed in a temporally distinct manner following brain injury. Specifically, at 6 h, females showed increased EP-3 receptor mRNA, and both sexes had higher EP-4 expression. Additionally, females, but not males had elevated EP-3 and EP-4 expression 24 h post-damage. Thus, we chose to antagonize these receptors during injury to test if these receptors are necessary for the induction of E_2_ using specific antagonists (L-798, 106, or BCG-20-1531 hydrochloride). Antagonism of prostanoid receptor(s) did prevent injury-induced E_2_ but in a sex-specific manner. Antagonism of EP-3 in males prevented E_2_ induction at 24 h, and antagonism of EP-4 prevented the induction in females at 6 h post-injury. These time points were chosen based on a previous study in our lab that found that inhibition of cox 1/2 signaling, and therefore PGE2, prevented the induction of E_2_ at 6 h for females and 24 h for males, and not vice versa. Given this data, we believe that PGE2 may bind to EP-3 in males and EP-4 in females to achieve the robust induction of E_2_ that has been well-documented following penetrating brain injury. However, additional doses of specific antagonists and/or time points are necessary to conclude that this represents a true sex-difference in the mechanism underlying the induction of aromatase by PGE2.

Prostanoid receptors have been shown to regulate aromatase in other systems in the periphery, including adipose stromal cells [28] and breast cancer cells [29] via EP-1, 2, or 4. These receptors have been shown to increase cAMP or intracellular calcium concentrations, which may induce aromatase and E_2_ content. In some systems, EP-3 decreases cAMP through G_i_ signaling [26]. However, it is unknown how these receptors work in the songbird brain. Our current data suggests that EP-3 and EP-4 may stimulate E_2_ following brain injury.

### Role of ERs in the anti-inflammatory effects of injury-induced E_2_

In order to determine how estrogen receptors change following brain injury, we measured three known receptors (ER-α, ER-β, and GPER1) using qPCR. Both males and females had elevated ERα and ERβ at multiple time points following injury, but we failed to detect changes in GPER1 at any time point. Thus, we chose to antagonize ER-α and ER-β with specific antagonists (MMP or PHTPP) to test if they are necessary for the reduction of PGE2 following brain injury. Previous work in our lab suggests that E_2_ induction acts as a potent anti-inflammatory signal [24]. Specifically, central E_2_ decreases cytokine and cox-2 mRNA, along with PGE2 content 24 h following penetrating brain injury. In the current study, antagonism of ER-α, and not ER-β, results in the prolonged elevation of PGE2 content. Thus, ER-α may be responsible for the anti-inflammatory actions of injury-induced E_2_; exploration of additional doses or time points may be necessary to understand this pathway fully.

Previous work has shown that ER-α is a potent anti-inflammatory signal following various types of brain insult [30–33, 59–61], but our data is the first to suggest that it may do so by decreasing PGE2 signaling in vivo. Although, similar in vitro work has identified an effect of E_2_ on PGE2 production [62], it seems to be mediated through ER-β [63]. Identification of the role of estrogen receptors play in regulation of PGE2 may have relevant implications from a therapeutic perspective. Selective estrogen receptor modulators (SERMs) may be appropriate for the treatment of neuroinflammatory disorders [64, 65]. The overexpression of COX-2 is prevalent in many neurodegenerative diseases or models of trauma, including epilepsy, Alzheimer’s disease, or ischemia [64]. SERMs can decrease of microglia activation [65] and have been used to limit inflammatory signaling in experimental models [65]. Our data suggest that ER-α may be necessary to limit excessive inflammatory signaling following damage and could be a potential therapeutic target.

## Conclusion

In summary, our data provides a mechanism of PGE2 induction of E_2_ following brain injury, and does so in a sex-specific manner. PGE2 binds to EP-3 in males and EP-4 in females to increase central E_2_ content. This induced E_2_ then decreases inflammatory signaling, and does so through ER-α. Our results show a newfound interaction between inflammatory signaling and estradiol synthesis.

## Abbreviations

ΔCT: Delta threshold cycle number
COX: Cyclooxygenase
E_2_: Estradiol
EP-r: E-prostanoid receptors
ER-r: Estrogen receptor
GPER: G-protein estrogen receptor
PGE2: Prostaglandin E2
Pik3: Phosphoinositide-3-kinase
SERM: Selective estrogen receptor modulators
TBI: Traumatic brain injury
